# Stability of Anthocyanins, Phenolics and Color of Tart Cherry Jams

**DOI:** 10.3390/foods8070255

**Published:** 2019-07-12

**Authors:** Josipa Vukoja, Anita Pichler, Mirela Kopjar

**Affiliations:** Department of Food Technologies, Faculty of Food Technology, Josip Juraj Strossmayer University, Franje Kuhača 20, 31000 Osijek, Croatia

**Keywords:** tart cherry jams, anthocyanins, phenol content, antioxidant activity, color, texture

## Abstract

The aim of this work was to assess the effect of a set storage period on the phenolics, anthocyanins, antioxidant activity, color, and texture of three types of jams made from tart cherry. The prepared samples of tart cherry jams were: (1) regular jam; (2) extra jam; and (3) light jam. The samples were stored at room temperature for eight months. Results of the investigated parameters after storage were compared with the results after sample preparations, and between the different jam samples. The light jam had the highest phenolic content and anthocyanin content (3.34 g/kg and 985.52 mg/kg). Consequently, the light jam had the highest antioxidant activity determined by ABTS (2,2’-azinobis(3-ethylbenzothiazoline sulfonic acid)) and DPPH (2,2-diphenyl-1-picrylhydrazil) method. After storage, the highest retention of the phenolics had regular jam and extra jam (85%), while the lowest retention (74%) were found in the light jam. Anthocyanin stability was the highest in the regular jam, then the extra jam (15%), and then the light jam, with retention of 22%, 15%, and 12%, respectively. Color parameters and textural parameters also depended on jam type, as well as on storage conditions. Since the investigated types of jams differed in content of fruit and water, these are the most important factors that cause the change in the retention of phenolics and anthocyanins, as well as a change in color and textural parameters.

## 1. Introduction

Tart cherries are a good source of phytochemicals, which strongly influence their quality, and contribute to its organoleptic attributes and nutritional value. Phenolics are one of the main groups of phytochemicals present in tart cherry fruits. Special attention has been focused on anthocyanins and polyphenols, which are responsible for red skin and fresh color, and possess strong antioxidant and anti-inflammatory properties [1,2,3]. There is a considerable demand for fresh fruit, as well as their products. Since many types of fruit are seasonal and their shelf life is limited, they must be processed to achieve stability over time. Processing may include preservation by several methods, such as processing into gel products (jams, jellies, marmalades), juice production, freezing, fermentation, and drying. Jams are very attractive and popular products among consumers. The jam recipe, processing procedures, storage conditions, and duration are important factors for jam quality. Jam formulation varies, due to the composition of the matrix ingredients that have an impact on the rheology of the produced jam. Any small alteration in the jam matrix (for example, replacing part of the sugar with other sweeteners, or using different pectins) can create changes in the food matrix constituents. As a consequence of changes in the interactions, jam quality will be dramatically affected [4]. Fruit jams are usually prepared, as described by Downing [5]. Traditionally jam is stored at room temperature in glass jars in warehouses and stores. Low temperature is generally not regarded as necessary to prevent degradation, as the jam during processing is added both preservatives and sugar, and the pH of the produce is usually low. The shelf life of jam is normally 6–12 months [6]. The storage of jam at room temperature for 1–3 months results in mild or moderate alterations of quality parameters, while more than six months of storage induced statistically significant alterations [7].

Quality parameters of jams, such as color, texture, total phenolics, antioxidant activity, and anthocyanins, can be affected by processing conditions, as well as storage conditions, thus, proper selection and balance of those parameters, next to ingredients selection, is of great importance for the production of high quality foods [8,9,10]. Anthocyanins, as a large phenolic group in tart cherry fruit, are stable under acid conditions, but under processing and storage conditions, they can transform into colorless compounds and insoluble brown pigments. Many factors can influence the stability of phenols and anthocyanins, like temperature, pH, light, oxygen, sugar, enzymes, presence of ascorbic acid, sulfite salts, metal ions and copigments [11,12,13,14,15].

The effect of fruit jam processing on antioxidant activity, total phenolics, and anthocyanins components has been previously evaluated. Kim and Padilla-Zakour [16] observed that thermal processing of the cherry fruits decreased the total phenolics, antioxidant capacity and total anthocyanin contents. Kopjar et al. [17] showed that pectin and storage time affected the color, antioxidant activity and texture of strawberry jams. Patras et al. [18] found that degradation of bioactive compounds, including ascorbic acid, anthocyanins, total phenolics, color, and antioxidant activity, occurred in strawberry jams during storage at both 4 °C and 15 °C. They also observed an increase in degradation of anthocyanins, color, and ascorbic acid with an increase in temperature. Oancea and Calin [19] showed that processing of blackberry, red raspberry, sweet cherry and wild cherry fruits into jams by a traditional method caused a significant change of the level of antioxidant compounds, in particular of anthocyanins which degraded by 66–84% compared to fresh fruits. The degradation of bioactive compounds continued during jam storage, but at slower rates.

Studies that focus on the behavior of total phenolics, antioxidant activity and anthocyanins of these fruit jams during long-term storage are rather limited. Furthermore, there is a need to increase our understanding of the effect of jam processing and its storage on nutraceutical contents. For these reasons, the effects of storage upon these compounds need to be investigated. The objective of the experiments was to investigate the stability of phenolics and anthocyanins, change of antioxidant activity, color, and texture of three types of jams: (1) Regular jam; (2) extra jam; and (3) light jam upon preparation and storage of eight months at room temperature (conditions as in-store).

## 2. Materials and Methods

### 2.1. Materials

Tart cherries were purchased at a local market, pits were removed, and flesh was used for the preparation of jams. The following chemicals were used: Potassium chloride, sodium acetate, hydrochloric acid, methanol, sodium carbonate, sodium bisulfite, Folin-Ciocalteu reagent were purchased from Kemika (Zagreb, Croatia). Trolox, 2,2’-azinobis (3-ethylbenzothiazoline sulfonic acid) (ABTS) and 2,2-diphenyl-1-picrylhydrazil (DPPH) were purchased from Sigma-Aldrich (St. Louis, MO, USA).

### 2.2. Preparation of Tart Cherry Jams

Three types of jam were prepared: (1) Regular jam; (2) extra jam; and (3) light jam. Formulations of all types of jams are given in Table 1. Jams were prepared by mixing fruit, water and a large part of sucrose. Citric acid was used for pH adjustment. Pectin was added at the final stage of jam cooking mixed with part of sucrose. The regular jam and extra jam were cooked until the final product contained 62.5% of soluble solids. Preparation time was 30 min at 85 °C. The light jam was cooked until the final product contained 41% of soluble solids. The time of cooking was 20 min at 85 °C.

### 2.3. Extraction of Phenols

1 g of the sample was extracted for 12 h with 10 mL of acidified methanol (methanol:HCl ratio was 98:2) at 4 °C [17]. After 12 h, the mixture was filtered and obtained extract was used for evaluation of total phenols, anthocyanins and antioxidant activity.

### 2.4. Determination of the Total Phenol Content

The total phenolic content in the samples was determined by Folin-Ciocalteu method [20]; briefly, 0.2 mL of extract and 1.8 mL of deionizer water were mixed with 10 mL (1:10) of Folin-Ciocalteu reagent and 8 mL of 7.5% solution of sodium carbonate. Prepared mixtures were left in the dark for 120 min to develop the color, and the absorbance was read at 765 nm by spectrophotometer (Cary 60 UV-Vis, Agilent Technologies, Santa Clara, CA, USA). For each sample, the measurements were performed in triplicates and values were interpolated on a gallic acid calibration curve and expressed as g of gallic acid equivalents per kg (g GAE/kg).

### 2.5. Measurement of Monomeric Anthocyanin Content and Polymeric Color

Determination of monomeric anthocyanin content and polymeric color were conducted by methods described by Giusti and Wrolstad [21]. Total anthocyanins were expressed as cyanidin-3-glucoside. Sample absorbance was read against a blank cell containing distilled water. Measurements were done in duplicates.

### 2.6. Calculation of Kinetic Parameters of Anthocyanin Degradation

The first-order reaction rate constants (k), half-lives (t_1/2_), i.e., the time which is necessary for degradation of 50% of anthocyanins, were calculated using the following equations:

ln (c_t_/c_0_) = −k × t,

t_1/2_ = −ln (0.5)/k, (1)

where c_0_ is initial anthocyanin content and c_t_ anthocyanin content after storage.

### 2.7. Antioxidant Activity

The ABTS assay followed the method of Arnao et al. [22] with some modifications. Briefly, 0.2 mL of extract was mixed with 3 mL of ABTS reagent and left in the dark. After 95 min, absorbance was measured at 734 nm. For blank, the extract was replaced with water. The antioxidant activity of the samples was also determined by the radical scavenging activity method using 2,2-diphenyl-1- picrylhydrazyl radical (DPPH) as, previously described by Brand-Williams et al. [23] with slight modification; 0.2 mL of extract was mixed with 2.8 mL of DPPH solution (0.5 mM) to the final volume of 3 mL. After 15 min, the absorbance was measured at 517 nm. Antioxidant activity evaluated by ABTS and DPPH method was calculated from the calibration curve with Trolox, as standard (µmol TE/100 g). All measurements were done in triplicate.

### 2.8. Color Measurement and Color Change

Color measurement and color change were monitored with a chromometer Minolta CR-30 (Minolta; Osaka, Japan). Lab system was used for evaluation of color. The established color parameters were as follows: L* (lightness—0 is black, and 100 is white); a* (redness (+) and greenness (−)); b* (yellowness (+) and blueness (−)); and C* (the color saturation value (chroma)), as well as h (the hue angle (from 0° for red, over 90° for yellow and 180° for green, up to 270° for blue and back to 0°)).

### 2.9. Texture Analysis

The texture analysis was performed directly in the jar at the ambient temperature with a Texture Analyzer TA.XT plus (Stable Mycro System, Godalming, UK), using back extrusion procedure. On the basis of the preliminary work, the instrument working parameters were determined with the test mode compression, pretest speed at 1 mm/s, test speed at 1 mm/s, post-test speed at 10 mm/s, distance 10 mm, trigger force at 10 g and data acquisition rate at 200 pp. The data were analyzed using Texture expert Version 1.22 Software (Stable Micro System, Godalming, UK) to measure the cohesiveness and viscosity index in the samples. The measurements were done in triplicates.

### 2.10. Statistical Analysis

Results were expressed as the mean values ± standard deviation. Data were analyzed by analysis of variance (ANOVA) and Fisher’s least significant difference (LSD) with the significance defined at *p* < 0.05. All statistical analyses were carried out using software program STATISTICA 13.1 (StatSoft Inc., Tulsa, OK, USA).

## 3. Results and Discussion

### 3.1. Total Phenolic Content

In Table 2, the total phenolic content in jams, after preparation and eight months of storage at the room temperature, are presented. The light jam had the highest content of total phenolics after preparation and after storage, in comparison to the other two jams. Total phenolic content in the light jam, after preparation was 3.34 g/kg, while extra jam and regular jam had 2.29 g/kg and 1.99 g/kg, respectively. After storage, the highest retention of total phenolic content was in the regular jam and extra jam, 85%. The lowest retention was at light jam (74%), probably due to lower sugar content and higher water content than in the jam and extra jam. This is in agreement with earlier reports showing that high-sugar jam contains higher total phenolic content compared with low sugar and light jams [24,25]. The concentration of monomeric anthocyanins (Table 2) in the light jam was the highest (986 mg/kg), followed by extra jam (572.8 mg/kg) and regular jam (437.39 mg/kg). After storage, monomeric anthocyanins decreased in all types of jam. Retention tendency was slightly different than for phenolics, highest in the regular jam (22%), then in the extra jam (15%), and light jam (12%). The highest retention of anthocyanins in the regular jam can be linked to sugar and water content. The attack by water converts the flavylium ion to colorless pseudo-base resulting in color loss. The positive effect of sugar on anthocyanin stability could be due to a decrease in water activity, since sugar molecules are effective in binding water molecules [26,27]. Additional positive effects of sugar could be assigned to its steric interference with condensation reaction products (anthocyanin-phenolic polymerization) [28], it can be partial oxygen barrier [28], and upon sugar addition, due to the removal of water displacement of the hydration/dehydration equilibrium toward the colored species [26] can occur.

After the preparation of the jams, the percent of polymeric color in all samples was around 24%, and after storage, this parameter increased in all samples. After storage, the percent of polymeric color in the regular jam was 45%, in the extra jam was 53% and in the light jam was 47% (Table 2) indicating anthocyanin polymers were formed in response to storage. These results were in agreement with other studies where an increase of percent of polymeric color occurred with losses of anthocyanins [29,30].

Stability of anthocyanin during storage, except for retention, was illustrated by the rate of degradation of anthocyanins and the half-life of anthocyanins (Table 3). Assuming that degradation of anthocyanins fits the first-order reaction model, as it was proven in many studies earlier [31,32,33,34], it was possible to calculate the reaction rate constants and half-life of anthocyanins degradation. The rate of degradation was highest in the light jam (0.258 months^−1^), slightly smaller in the extra (0.231 months^−1^), and the lowest in the regular jam, 0.186 months^−1^. The half-life of anthocyanin is the largest in the regular jam, 3.72 months, then in the extra jam, 2.99 months, and in the light jam for 2.68 months.

### 3.2. Total Antioxidant Activity

The antioxidant activity was determined by ABTS and DPPH methods. Results of determination of the antioxidant activity of tart cherry jams are presented in Table 4. The tendency of results obtained from those two methods was slightly different. After preparation, results for DPPH method were 4.149 µmol TE/100 g, 4.388 µmol TE/100 g and 4.78 µmol TE/100 g for regular, extra and light tart cherry jams, respectively. Values for antioxidant activity obtained by ABTS method were similar for regular and extra jam (around 3.7 µmol TE/100 g) and much higher for light jam, 5.662 µmol TE/100 g. The light jam had the highest antioxidant activity according to both used methods. During storage, there was a decrease in antioxidant activity, but not so much as in the case of the phenols and anthocyanins, the compounds responsible for the antioxidant activity of the jams. The regular and extra jam had the same antioxidant activity after storage, while light jam had much higher values when the DPPH method was applied. Results of the ABTS method showed that antioxidant activity increased as followed regular jam, extra jam and light jam. Interestingly in the case of extra jam, there was no significant change of antioxidant activity after preparation and after storage. The loss of antioxidant activity resulting from the reduction of the phenol content is most likely compensated by the formation of oxidized and/or polymerized phenols, which may have higher antioxidant activity than non-oxidized phenols. In addition, the formation of Maillard products also can contribute to antioxidant properties. In the case of light jam antioxidant activity after preparation and after storage was higher using ABTS free radicals. In the case of the other two jams, a different tendency has been observed. In the regular jam sample, higher antioxidant activity after preparation and storage was determined by the application of DPPH radicals. The differences obtained in the results of the antioxidant activity are because different methods have been used—in which different free radicals are used for the determination of the antioxidant activity, and different reactions between free radicals and antioxidants in the samples vary, depending on the structure of phenolic substances [12]. ABTS radicals have poor selectivity in reactions with hydrogen donors because it reacts with any aromatic compound with a hydroxyl group, regardless of its actual antioxidant potential [35,36,37]. Unlike ABTS, DPPH radicals do not react with flavonoids which do not have a hydroxyl group on the B ring, nor with aromatic acids having only one hydroxyl group [37,38].

### 3.3. Color

Color is one of the fundamental criteria for the visual assessment of jams. Results of measurement of color parameters L*, a*, b*, C and °h of regular jam, extra jam and light jam are presented in Table 5. Immediately after preparation, the L* parameter determining color brightness was between 25.38 and 25.71. In light jam, L* value increased after storage conditions, but in jam and extra jam L* value decreased. Values for redness parameter (a*) were higher after preparation in comparison with jam samples after storage, where a* values decreased. These indicate that the jam during storage conditions lost its particular color, reducing lightness and changing from initial the most redish (highest a*) value to yellowish tones. This can also be linked to a decrease in anthocyanins content and formation of brown pigment by Maillard reaction [39]. The b* values also decreased in a similar way, and were significantly affected by jam processing. The lowest value of the C* parameter was determined in jam (1.49) and the highest in the light jam (3.29) after preparation. After storing at room temperature for eight months, the C* value (color saturation) changed in all types of jam. The value of the °h parameter was within the red coloration range (258.97-355.5).

### 3.4. Texture

Textural parameters that were determined were cohesiveness and viscosity index. They were different between all types of jams during preparation and storage (Table 6). Cohesiveness represents the strength of the internal bonds making up the body of the product. It is expected to be inversely proportional to the rate at which the material fractures under mechanical action [40]. Cohesiveness was the highest in the light jam after preparation, which had the lowest concentration of pectin. Abid et al. [41] showed that the effect of increasing pectin concentration decreased cohesiveness. This means that jams fractured more easily with increasing pectin concentration. After storage, cohesiveness increased in the regular jam (27%) and decreased in the extra jam and light jam, by 6% and 13%, respectively, when compared to the samples after preparation. After preparation, the highest viscosity index had light jam, followed by extra jam and regular jam. All types of jam showed different tendency after the storage. Viscosity index increased in the regular jam by 13%, in the extra jam remained similar, and decreased by 12% in the light jam.

## 4. Conclusions

In this study, the content of total phenols, anthocyanins and antioxidant activity, color, and texture parameters of three types of tart cherry jams, after preparation and after storage at room temperature for eight months, were evaluated. The light jam was found to have the highest content of total phenols, as well as content of anthocyanins after preparation; then the extra jam, and finally, the regular jam. After storage, the content of phenols and anthocyanins decreased in all jam types, but the highest stability of phenols and anthocyanins was observed in the regular jam.

## Figures and Tables

**Table 1:** Formulations of tart cherry jams.

	Regular Jam	Extra Jam	Light Jam
Fruit	700 g	700 g	1000 g
Water	180 g	140 g	-
Sucrose	850 g	730 g	300 g
Pectin	10 g	8 g	6 g

**Table 2:** Total phenolic, monomeric anthocyanins content and polymeric color of regular jam, extra jam and light jam after preparation and storage at room temperature for eight months.

Samples		Phenolic Content (g GAE/kg)	Monomeric Anthocyanins Content (mg cya-3-glu/kg)	Polymeric Color (%)
Regular jam	Preparation	1.99 ± 0.06 ^b^	437.39 ± 1.25 ^d^	24.68
	Storage	1.69 ± 0.14 ^a^	98.48 ± 1.01 ^b^	44.93
Extra jam	Preparation	2.29 ± 0.07 ^c^	572.8 ± 1.54 ^e^	24.85
	Storage	1.95 ± 0.18 ^b^	89.71 ± 1.11 ^a^	53.56
Light jam	Preparation	3.34 ± 0.09 ^d^	985.52 ± 1.49 ^f^	24.10
	Storage	2.48 ± 0.11 ^c^	124.14 ± 1.14 ^c^	47.71

Means ± standard deviation in the same column with the same letters are not significantly different (*p* ≤ 0.05). (cya-3-glu-cyanidin-3-glucoside; GAE—gallic acid equivalent).

**Table 3:** Rate of degradation of anthocyanins (k) and time of half-life of anthocyanins (t_1/2_) in the regular, extra and light tart cherry jams after storage.

Samples	k (Months^−1^)	t_1/2_ (Months)
Regular jam	0.186371	3.72
Extra jam	0.231739	2.99
Light jam	0.258970	2.68

**Table 4:** Antioxidant activity of jam, extra jam and light tart cherry jams after preparation and after storage at room temperature for eight months.

Samples		DPPH (μmol TE/100 g)	ABTS (μmol TE/100 g)
Regular jam	Preparation	4.149 ± 0.055 ^b^	3.656 ± 0.047 ^b^
	Storage	3.563 ± 0.087 ^a^	3.251 ± 0.036 ^a^
Extra jam	Preparation	4.388 ± 0.039 ^c^	3.723 ± 0.018 ^b^
	Storage	3.669 ± 0.015 ^a^	3.687 ± 0.027 ^b^
Light jam	Preparation	4.78 ± 0.038 ^d^	5.662 ± 0.029 ^c^
	Storage	4.281 ± 0.031 ^b^	5.302 ± 0.039 ^c^

Means ± SD in the same column with the same letters are not significantly different (*p* ≤ 0.05) (TE—Trolox equivalent).

**Table 5:** Color parameters (L*, a*, b*, C* and °h) of regular, extra and light tart cherry jams after preparation and after storage at room temperature for eight months.

Samples		L*	a*	b*	C*	°h
Regular jam	Preparation	25.42 ± 0.08 ^a^	1.46 ± 0.04 ^b^	−0.3 ± 0.02 ^d^	1.49 ± 0.09 ^b^	348.2 ± 0.1 ^d^
	Storage	25.33 ± 0.06 ^a^	0.97 ± 0.02 ^a^	−0.32 ± 0.04 ^d^	1.12 ± 0.08 ^a^	343.0 ± 0.2 ^c^
Extra jam	Preparation	25.38 ± 0.07 ^a^	1.98 ± 0.02 ^c^	−0.13 ± 0.01 ^b^	1.72 ± 0.07 ^c^	355.5 ± 0.2 ^f^
	Storage	25.26 ± 0.07 ^a^	1.51 ± 0.04 ^b^	−0.23 ± 0.04 ^c^	1.54 ± 0.08 ^b^	352.9 ± 0.1 ^e^
Light jam	Preparation	25.71 ± 0.05 ^b^	3.76 ± 0.03 ^e^	0.23 ± 0.03 ^e^	3.26 ± 0.06 ^e^	290 ± 0.2 ^b^
	Storage	26.04 ± 0.09 ^c^	2.81 ± 0.04 ^d^	−0.04 ± 0.01 ^a^	2.88 ± 0.09 ^d^	259 ± 0.2 ^a^

Means ± SD in the same column with the same letters are not significantly different (*p* ≤ 0.05).

**Table 6:** Parameters of the texture of regular, extra and light tart cherry jams after preparation and after storage at room temperature for eight months.

Samples		Cohesion (g)	Viscosity Index (gs)
Regular jam	Preparation	121.56 ± 2.32 ^a^	60.21 ± 2.11 ^a^
	Storage	154.07 ± 2.65 ^b^	69.05 ± 2.41 ^b^
Extra jam	Preparation	178.98 ± 2.45 ^d^	70.22 ± 2.65 ^b^
	Storage	168.34 ± 2.36 ^c^	73.52 ± 2.18 ^b^
Light jam	Preparation	219.19 ± 1.99 ^f^	93.7 ± 2.23 ^d^
	Storage	190.63 ± 2.41 ^e^	82.52 ± 2.11 ^c^

Means ± SD in the same column with the same letters are not significantly different (*p* ≤ 0.05).
